# Taft's Operative Dentistry

**Published:** 1860-01

**Authors:** 


*Taft's Operative Dentistry.*—This handsome book, which has now been before the profession for some months, was received just as our last number had issued from the press, and since then ill health has prevented us from reading it, and forming an opinion as to its merits. We have no doubt, however, that the reader will derive advantage from so thorough a study of any branch of his profession as the treatise on operative dentistry before us appears likely to afford.

